# Transcriptomic Analysis During Olive Fruit Development and Expression Profiling of Fatty Acid Desaturase Genes

**DOI:** 10.3390/ijms252011150

**Published:** 2024-10-17

**Authors:** Alicia Serrano, Judith García-Martín, Martín Moret, José Manuel Martínez-Rivas, Francisco Luque

**Affiliations:** 1 The University Institute of Research in Olive Grove and Olive Oils (INUO), University of Jaén, 23071 Jaén, Spain; jgm00117@red.ujaen.es (J.G.-M.); mmoret@ujaen.es (M.M.); 2 Instituto de la Grasa, CSIC (Consejo Superior de Investigaciones Científicas), 41013 Seville, Spain; mrivas@ig.csic.es

**Keywords:** *Olea europaea* L., olive fruit, ripening process, RNAseq, fatty acid desaturase

## Abstract

The olive fruit is a drupe whose development and ripening takes several months from flowering to full maturation. During this period, several biochemical and physiological changes occur that affect the skin color, texture, composition, and size of the mesocarp. The final result is a fruit rich in fatty acids, phenolic compounds, tocopherols, pigments, sterols, terpenoids, and other compounds of nutritional interest. In this work, a transcriptomic analysis was performed using flowers (T0) and mesocarp tissue at seven different stages during olive fruit development and ripening (T1–T7) of the ‘Picual’ cultivar. A total of 1755 genes overexpressed at any time with respect to the flowering stage were further analyzed. These genes were grouped into eight clusters based on their expression profile. The gene enrichment analysis revealed the most relevant biological process of every cluster. Highlighting the important role of hormones at very early stages of fruit development (T1, Cluster 1), whereas genes involved in fatty acid biosynthesis were relevant throughout the fruit developmental process. Hence, genes coding for different fatty acid desaturase (SAD, FAD2, FAD3, FAD4, FAD5, FAD6, and FAD7) enzymes received special attention. In particular, 26 genes coding for different fatty acid desaturase enzymes were identified in the ‘Picual’ genome, contributing to the improvement of the genome annotation. The expression pattern of these genes during fruit development corroborated their role in determining fatty acid composition.

## 1. Introduction

The olive tree (*Olea europaea* L.) is cultivated for the nutritional value of its fruit, a drupe consisting of a fleshy mesocarp and a woody endocarp. The development and ripening of the olive fruit is a long process lasting several months, which could be divided into five phases [1]. The development process starts immediately after the pollination and fruit set, following a double sigmoid growth curve pattern [2]. The first phase is characterized by an exponential growth of the embryo due to a rapid cell division that usually lasts around half a month after flowering [3]. During the following 30 days, a phase of cell division and expansion takes place, leading to the mesocarp growth and endocarp development. Pit hardening concurs with a recession of cell division and a reduction in fruit growth. Subsequently, the green fruit continues growing until it reaches the final size by cell expansion at the yellowish stage [4]. Finally, the ripening phase leads to changes to the purple color of the skin, from the turning stage or veraison to completely purple or black.

The length of the fruit development and ripening process exhibits variations among cultivars [4], and the final composition can differ significantly, even during fruit development [2]. In any case, olive fruit has unique characteristics due to its composition, including fatty acids, phenolic compounds, tocopherols, pigments, sterols, and terpenoids. These compounds are transferred to virgin olive oil, giving it a unique flavor and healthy properties [5]. The biosynthesis of these compounds involves several biochemical and physiological changes during olive fruit development [1]. Despite the efforts made to understand the molecular mechanisms underlying these metabolic processes, numerous genes involved in their biosynthetic pathways are still unknown [6].

Transcriptomic studies focused on fatty acid biosynthesis have been reported in the cultivars ‘sylvestris’ [7], ‘Leccino’ [8], ‘Arbequina’, ‘Frantoio’, and ‘Nikitskii’ [9], whereas in the ‘Picual’ cultivar are mainly related to fruit abscission [10,11,12], early fruit development [3], and hormonal control [13].

Regarding the oil content, it accumulates mainly in the mesocarp after pit hardening and reaches its maximum during veraison, representing up to 30% of the fresh weight of the ripe fruit [1,14]. The most abundant fatty acid is oleic acid (C18:1), which represents about 75% of total fatty acids, followed by linoleic (C18:2), palmitic (C16:0), stearic (C18:0), and linolenic (C18:3) acids. The pattern of fatty acid biosynthesis and desaturation varies during fruit development and between cultivars, with a higher proportion of oleic than linoleic acid in the final content being desirable [15]. Therefore, a thorough understanding of the fatty acid biosynthesis process during fruit development could contribute to producing higher-quality oils.

Four genes encoding for stearoyl–ACP desaturase (*SAD*) enzyme have been described in olive involved in oleic acid formation: *OeSAD1* [16], *OeSAD2*, *OeSAD3* [17], and *OeSAD4* [18]. Moreover, five genes have been characterized as encoding for microsomal oleate desaturases (*OeFAD2-1*, *OeFAD2-2*, *OeFAD2-3*, *OeFAD2-4*, *OeFAD2-5*) [19,20,21,22], which are responsible for linoleic acid content together with the plastidial isoform *OeFAD6* [19,23]. Similarly, genes involved in the biosynthesis of linolenic acid have been studied in olive, including four linoleate desaturases. Two are located in the endoplasmic reticulum: *OeFAD3A* [24] and *OeFAD3B* [25]. Another two possess plastidial localization: *OeFAD7-1* [26] and *OeFAD7-2* [25]. In addition, genes corresponding to FAD4 [27] and FAD5 [28] have not been studied in olive. The expression of genes coding for most of these enzymes and isoforms has been further analyzed in several cultivars with low and high linoleic acid content [15], but, up to date, there are no transcriptomic studies encompassing all these genes during fruit development.

This work represents an overview of the genes overexpressed throughout fruit development, from flower to ripe fruit mesocarp. We analyze their expression profiles and the biological processes in which they are involved in order to identify relevant genes for the biosynthesis of olive fruit compounds. Moreover, in the ‘Picual’ genome, this work aims to identify genes coding for fatty acid desaturases, which could contribute to improving the genome annotation, and their expression was analyzed throughout fruit development from flower to ripened fruit. In short, this work aims to provide a better understanding of the molecular mechanism involved in fruit development, which could be crucial for molecular marker development.

## 2. Results

### 2.1. Gene Expression During Fruit Development

**Figure 1:**
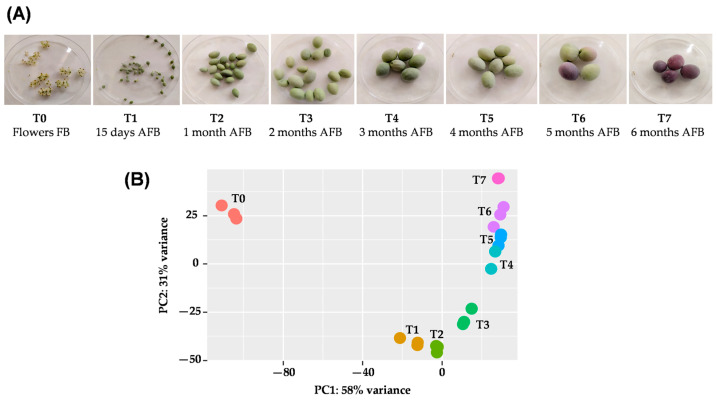
(**A**) Developmental stages collected for RNAseq analysis. (**B**) PCA plot showing the expression differences among olive fruit developing samples. Samples collected in triplicate: T0: flowers at full bloom, T1: fruits at 15 days after full blooming (AFB), T2: fruits at 1 month AFB, T3: fruits at 2 months AFB, T4: fruits at 3 months AFB, T5: fruits at 4 months AFB, T6: fruits at 5 months AFB, and T7: fruits at 6 months AFB.

The development of the olive fruit is a process that takes around 6 months from full bloom to fruit ripening (Figure 1A).

In this work, different gene expressions were observed at each sampling time throughout fruit development. The flowering stage (T0) clearly differed from the rest of the fruit stages, and samples collected at 0.5, 1, and 2 months after full bloom (AFB) (T1, T2, and T3, respectively) and corresponding to early developmental stages of the whole fruit are significantly distinct from the other mesocarp samples from fruit collected at 3, 4, 5, and 6 months AFB (T4, T5, T6, and T7, respectively) (Figure 1B). The percentages of read mapping to the reference genome ranged from 74.3% to 78.7% (Table S1).

**Figure 2:**
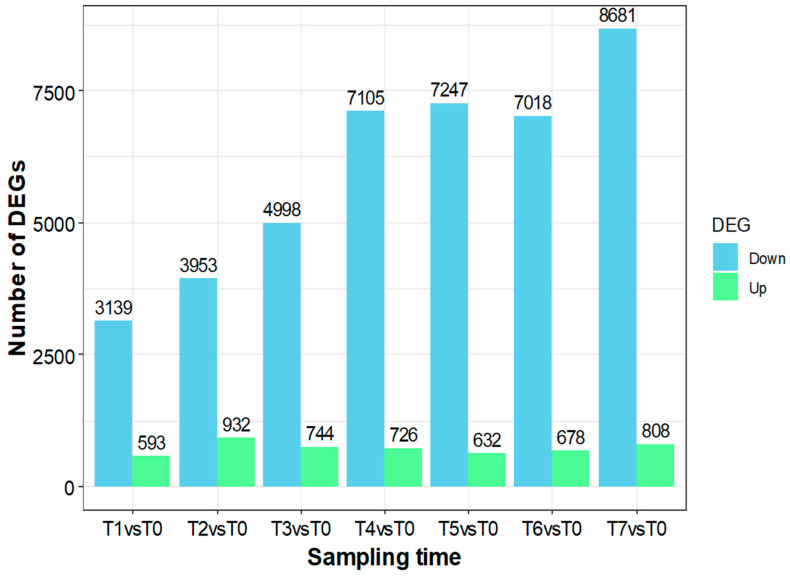
Differentially expressed genes throughout fruit development owing to the flowering stage. T0: flowers at full bloom, T1: 15 days after full blooming (AFB), T2: 1 month AFB, T3: 2 months AFB, T4: 3 months AFB, T5: 4 months AFB, T6: 5 months AFB, and T7: 6 months AFB.

Thus, the flowering stage (T0) was considered the reference time for the analysis of the differential gene expression throughout the fruit development from 15 days to 6 months AFB (T1–T7) (Figure 2). The results pointed out a predominance of repressed genes (Down) along the entire olive fruit development process, which means that more genes are overexpressed in the flowering stage. Likewise, the number of repressed genes in fruits increases throughout fruit development, showing the highest value when the fruit is fully mature at T7 (6 months AFB). The number of overexpressed genes in fruit ranged from 539 at 15 days after flowering (T1) to 932 one month after flowering (T2). Overall, 13,486 differentially expressed genes were identified with a *p*-adj value < 0.01 and a fold change higher than 4. Of those, 2,160 genes were overexpressed at any time during fruit development compared to the flowering stage. The expression profile of those genes overexpressed at some time with respect to the flowering stage and with an expression value (log_2_CPM) higher than 0 was analyzed with DPGP v0.1 software [29].

Genes overexpressed at any stage of fruit development received special attention because of their potential role in interesting biological processes in olive fruit. In total, 1755 genes were gathered into 37 clusters according to their expression trend along the fruit development (Figure S1). However, clusters showing similar trends were reorganized into eight clusters (A–H), excluding those clusters showing a random expression pattern during fruit development (clusters 12, 33, 36, and 37 from DPGP). Genes included in each cluster, along with their annotation, are available in the Supplementary Information (Table S2). The eight clusters revealed several GO terms related to biological processes characteristic of fruit development (Table S3). In the following cluster description, only the 20 most representative processes according to the FDR and fold enrichment values obtained in ShinyGO 0.8 will be presented.

**Figure 3:**
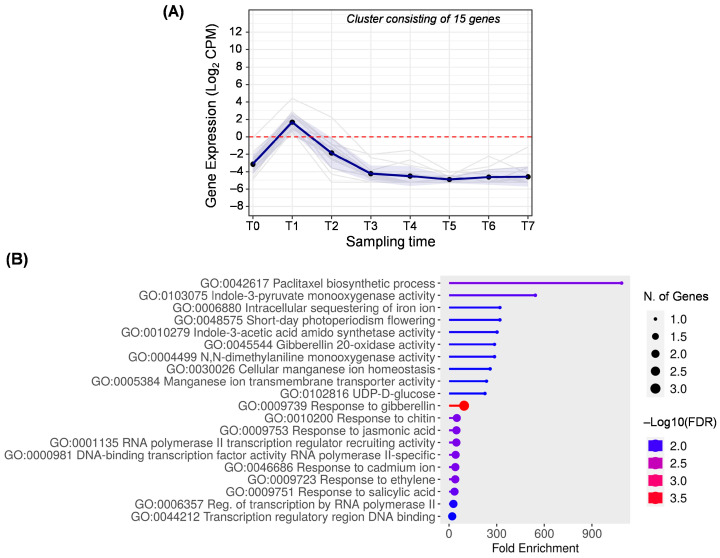
Cluster A. (**A**) Gene expression profile. Blue line represents the mean value of expression for the total genes in the group. Blue shadow represents the standard error of gene expression. Gray lines represent the expression of individual genes. The red horizontal line represents the threshold separating positive from negative expression levels. (**B**) The 20 most representative biological processes according to the FDR and fold enrichment values obtained in ShinyGO 0.80.

Cluster A (Figure 3) corresponds to cluster 35 obtained in DPGP and includes 15 overexpressed genes at T1, i.e., 15 days AFB. Interestingly, the most enriched term in this cluster is related to the synthesis of paclitaxel (GO:0042617), a diterpenoid noted for its anticancer properties [30]. This compound has not been previously described in olive since terpenes have been studied in advanced stages of fruit development, representing a minority percentage among the volatile compounds, and their content decreases as the fruit ripens [31,32,33]. However, the terpenoids studied in olive fruit are those coming from the phenylpropanoid pathway, which acts to the detriment of the paclitaxel synthesis pathway [30].

The functional enrichment analysis also revealed genes related to the hormone response, such as auxin, gibberellin, jasmonic acid, ethylene, and salicylic acid, which are very important in plant growth and development. Two GO terms related to the synthesis of indole-3-acetic acid (GO:0103075, GO:0010279), the main auxin in plants, and two GO terms related to gibberellin (GO:0045544, GO:0009739) were detected. Both hormones play a crucial role in the fruit set and in cell division in the early stages of fruit development [3,34]. For instance, in tomato and melon, an overexpression of auxin synthesis-related genes has been observed in the early stages of fruit development, coinciding with an accumulation of indole-3-acetic acid, decreasing as the ripening process progresses [35,36]. Likewise, *N*,*N*-dimethylaniline monooxygenase (GO:0004499) is a flavin monooxygenase, as well as indole-3-pyruvate monooxygenase (GO:0103075). Both compounds influence the natural synthesis of gibberellin and auxins [36]. Among others, enriched terms related to micronutrients required for fruit development, such as iron and manganese, also appeared in cluster A [37].

**Figure 4:**
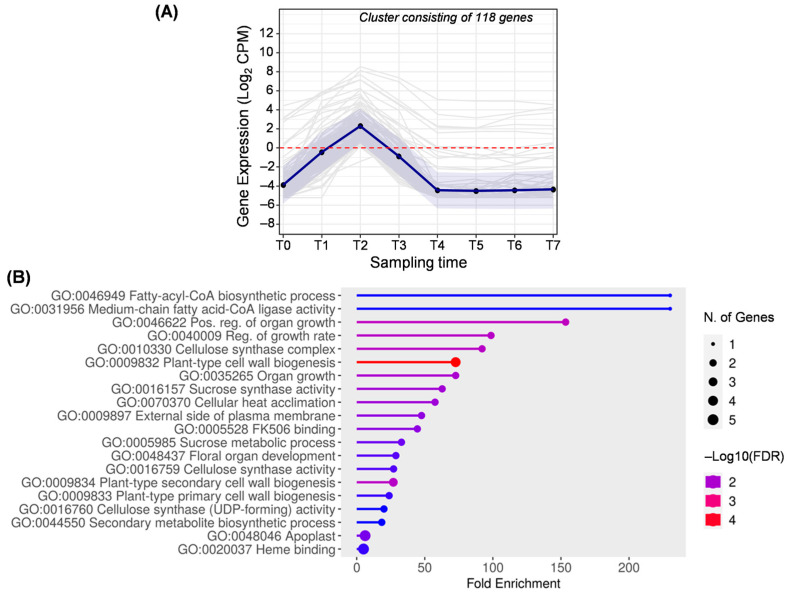
Cluster B. (**A**) Gene expression profile. Blue line represents the mean value of expression for the total genes in the group. Blue shadow represents the standard error of gene expression. Gray lines represent the expression of individual genes. The red horizontal line represents the threshold separating positive from negative expression levels. (**B**) The 20 most representative biological processes according to the FDR and fold enrichment values obtained in ShinyGO 0.80.

The second cluster (Figure 4) was the result of merging clusters 20 and 34 generated by the DPGP algorithm (Figure S1), including a total of 118 overexpressed genes at T2 (1 month AFB). Enrichment analysis revealed GO terms related to acyl–CoA biosynthesis (GO:0046949) and medium-chain fatty acid–CoA ligase activity (GO:0031956), which are related to lipid biosynthesis. At T2, olive fruits were actively growing, which is reflected in the enrichment of growth-related GO terms (GO:0046622, GO:0040009, GO:0035265, GO:0048437) and the close terms GO:0020037 and GO:0044550. Genes with the term GO:0020037 (heme-binding) belong to the cytochrome P450 family, and others are annotated as peroxides (Table S2). Specifically, peroxidases have been particularly induced during the early stage of olive fruit growth [38]. The genes enriching the term GO:0048437 are genes annotated as *CYP78A5*, a cytochrome P450 protein, which have been shown to stimulate cell proliferation and promote seed growth [39].

At this early stage of fruit development, genes involved in cellulose synthesis and cell wall biogenesis (GO:0010330, GO:0016759, GO:0016760, GO:009832, GO:009833, and GO:009834) are also prominent, supporting the fruit growth. In addition, enriched terms related to sucrose metabolism appeared (GO:0005985 and GO:0016157), which is consistent with previous studies that also observed overexpression of genes encoding enzymes related to starch and sucrose synthesis at 30 days AFB [2]. This makes sense, as olive mesocarp cells require sugars to synthesize oil [40]. In addition, the enrichment analysis revealed genes related to the heat-stress response (GO:0070370 and GO:0005528), which could affect fruit development, subsequent fruit yield, and oil quality [41,42].

**Figure 5:**
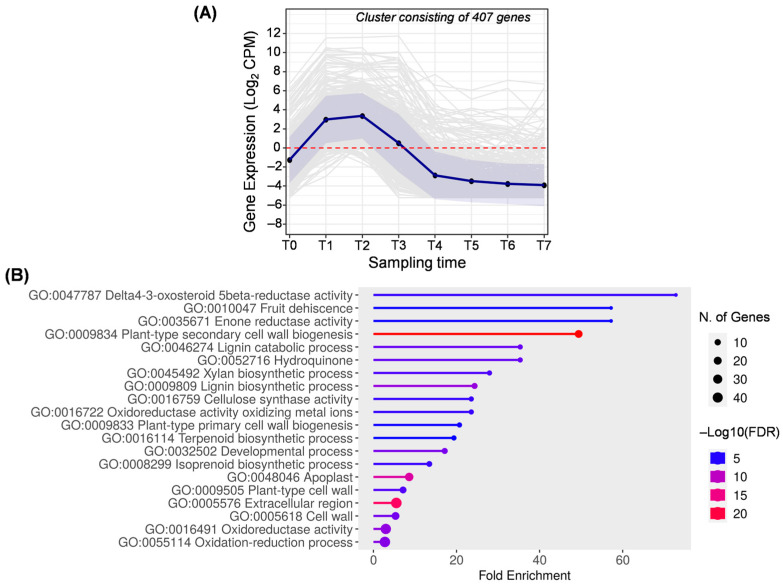
Cluster C. (**A**) Gene expression profile. Blue line represents the mean value of expression for the total genes in the group. Blue shadow represents the standard error of gene expression. Gray lines represent the expression of individual genes. The red horizontal line represents the threshold separating positive from negative expression levels. (**B**) The 20 most representative biological processes according to the FDR and fold enrichment values obtained in ShinyGO 0.80.

Cluster C (Figure 5) consists of 407 genes overexpressed during the early stages of fruit development, including T1, T2, and T3. This period encompasses the process of cell division and expansion until pit hardening (at T3, the pit was hardened). In this case, some biological processes had already been highlighted in clusters A and B, as they are related to cell division and expansion (GO:0009833, GO:0009834, GO:0009505, and GO:0016759). In addition to cellulose, this cluster includes the term GO:0045492 for xylan biosynthesis, which is also related to cell wall biogenesis. Xylan is a hemicellulosic polysaccharide abundant in the cell wall of olive fruit pulp [43].

The presence of genes related to cellulose and xylan compounds in clusters A and B supports the hypothesis that those compounds are more abundant in unripe fruit and are degraded along the ripening process [44]. Moreover, cell wall polysaccharides are the main factors responsible for fruit softening during ripening [43,44], and remodeling of these compounds along with the lignification could be involved in fruit abscission [45]. This fact may explain the appearance of the lignin process (GO:0046274 and GO:009809) and fruit dehiscence (GO:0010047) together in that group. The early fruit drop may be due to competition of the fruits for nutrients [46]. In the ‘Picual’ cultivar, the premature fruit drop has been previously observed at 217 days AFB and is characterized by an increase in the expression of genes from the *MYB* and *bZIP* (basic leucine zipper) families, as well as a higher accumulation of sterols [10]. Despite the occurrence of premature fruit drops, fruit drops are genetically programmed to occur when the fruit is mature.

The most enriched biological process in cluster C was related to steroid biosynthesis (GO:0047787), which are health-promoting compounds. In previous studies on fruits of ‘Coratina’ and ‘Tendellone’ olive cultivars, transcripts related to steroid synthesis were also detected in the early stages of fruit development [47]. Generally, this group includes various oxidoreductases (GO:0047787, GO:0035671, GO:0016722, GO:0016722, GO:0016491, and GO:0055114), which can act at different levels of the phenylpropanoid pathway. Specifically, two of them (GO:0016722 and GO:0052716) are correlated with lignin biosynthesis (GO:0009809 and GO:0046274). Lignin deposition and cellulose biosynthesis are essential for pit hardening [4], which occurred between T2 and T3 in this study. In this early stage of fruit development, genes involved in the synthesis of isoprenoids and terpenoids (GO:0008299 and GO:0016114) are also prominent.

**Figure 6:**
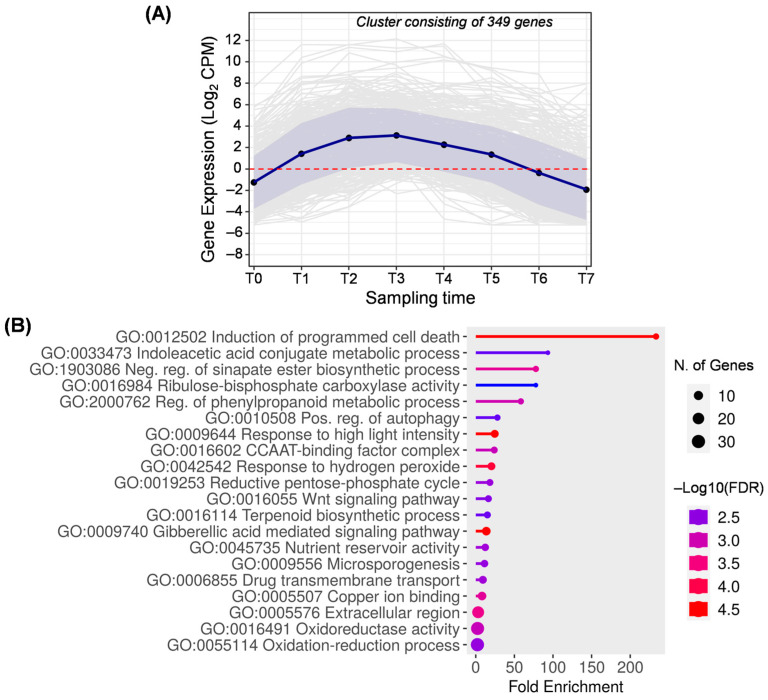
Cluster D. (**A**) Gene expression profile. Blue line represents the mean value of expression for the total genes in the group. Blue shadow represents the standard error of gene expression. Gray lines represent the expression of individual genes. The red horizontal line represents the threshold separating positive from negative expression levels. (**B**) The 20 most representative biological processes according to the FDR and fold enrichment values obtained in ShinyGO 0.80.

The result of grouping 349 genes that are overexpressed between T1 and T5 is the cluster D (Figure 6), which also corresponds to the union of clusters 9, 11, 16, 21, 24, 25, and 30 generated by DPGP (Figure S1). This group is mainly characterized by a peak of expression between T2 and T3 when pit hardening takes place. These results are in line with previous work confirming that programmed cell death (GO:0012502) and secondary thickening of the cell wall are characteristic of stone cell formation in drupes, as well as the deposition of cellulose and lignin [4,48]. In this line, genes related to the phenylpropanoid pathway and lignin synthesis (GO:1903086 and GO:2000762) also stood out in cluster D (Figure 6). Although they could also be related to cell death processes, this cluster includes genes involved in autophagy (GO:0010508) and response to high light intensity (GO:0009644). However, it is more likely that these mechanisms are associated with the response to abiotic stress.

In these intermediate stages, fruit development is also regulated through genes involved in the conjugation of indole-3-acetic acid (GO:0033473). This auxin, together with gibberellins (GO:0009740), is important for olive fruit development [13], supporting previous findings in different plant species in which both hormones have an important role in the regulation of cell division and expansion in fruits [34]. In olives, gibberellins have been suggested to regulate fruit size and the progression of the ripening process [13].

Between T1 and T5, olive fruits remained green, so they had active chloroplasts performing photosynthesis. This fact is supported by the enriched biological process related to the Ribulose–bisphosphate carboxylase activity (GO:0016984), which can be involved in fruit photosynthesis, providing additional sugars and organic compounds necessary to be used for fruit development and fatty acid biosynthesis in the olive mesocarp [40,49]. Related to this enzymatic activity, genes involved in the biological process GO:0019253 (reductive pentose–phosphate cycle) have also been detected.

Cluster D is also enriched in CCAAT-binding factors (GO:0016602), which are transcription factors involved in several processes, highlighting their role in determining the flowering time, seed maturation, and fatty acid biosynthesis [50,51]. These transcription factors interact with gibberellic acid and abscisic acid, also playing important roles in flavonoid biosynthesis, photomorphogenesis, photosynthesis, response to stress, and reproductive development [50].

**Figure 7:**
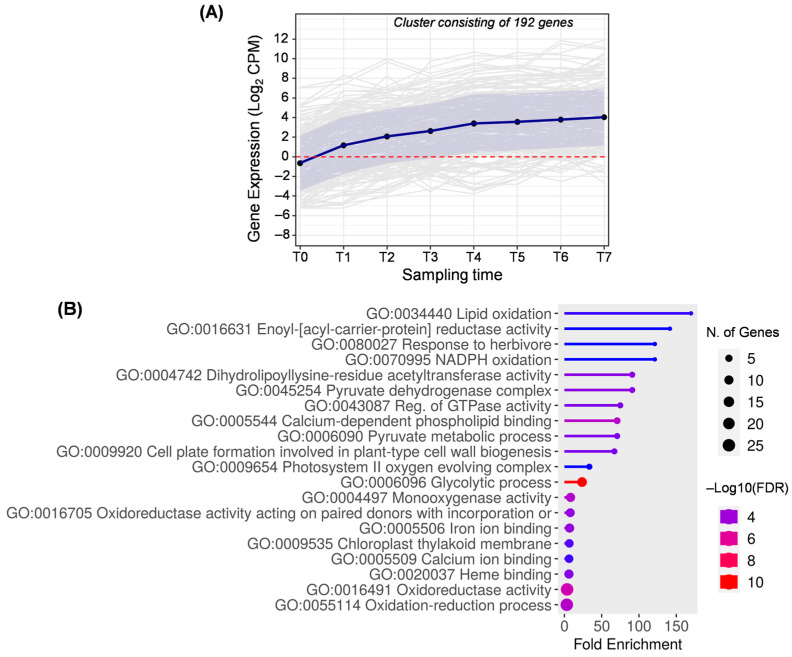
Cluster E. (**A**) Gene expression profile. Blue line represents the mean value of expression for the total genes in the group. Blue shadow represents the standard error of gene expression. Gray lines represent the expression of individual genes. The red horizontal line represents the threshold separating positive from negative expression levels. (**B**) The 20 most representative biological processes according to the FDR and fold enrichment values obtained in ShinyGO 0.80.

Cluster E includes 192 genes that are overexpressed relative to the flowering stage, with an upward trend as the development process progresses (Figure 7). This cluster is the result of joining clusters 2, 6, 15, 18, and 22 generated by DPGP (Figure S1).

Genes involved in lipid oxidation are noted in cluster E, but they are genes annotated as lipoxygenase 2 (LOX2) (Table S2), which produce mainly 13-hydroperoxides from linoleic and linolenic acids [52]. Previous studies about lipoxygenase activity in ‘Picual’ olive fruits also described the upregulation of LOX2 during early fruit development [3]. Lipoxygenase enzymes are responsible for volatile compounds which contribute to olive oil aroma and can also act as resistance mechanisms [53]. For this reason, the biological process of response to herbivores (GO:0080027) is also in this cluster.

This cluster has a broad representation of genes involved in the first steps of the fatty acid synthesis pathway, starting with the photosynthetic process. The photosystem II (GO:0009654) provides NADP which is reduced to NADPH (GO:0070995) necessary for fatty acid biosynthesis. Hence, the glycolytic process (GO:0006096) involves the breakdown of carbohydrates into pyruvate with the concomitant reduction in NADP to NADPH (GO:0006090) [54]. Pyruvate is required for acetyl–CoA biosynthesis through the activity of the pyruvate dehydrogenase complex (GO:0045254) [8,49]. The dihydrolipoyllysine-residue acetyltransferase (GO:0004742) is the E2 component of the pyruvate dehydrogenase complex (GO:0045254) and is essential for the oxidative decarboxylation of pyruvate to acetyl–CoA [55]. These two metabolites are considered the main precursors for fatty acid biosynthesis. Additionally, the enoyl-[acyl-carrier-protein] reductase (GO:0016631) also uses NADPH as co-factor for enoyl–ACP reduction resulting in acyl–ACP [54,56]. This enzyme belongs to the enzymatic complex fatty acid synthase (FAS), and it has been characterized in olive fruit in previous studies, showing a similar expression pattern [57]. Proteomic studies described a peak of accumulation around 110 days AFB [49].

**Figure 8:**
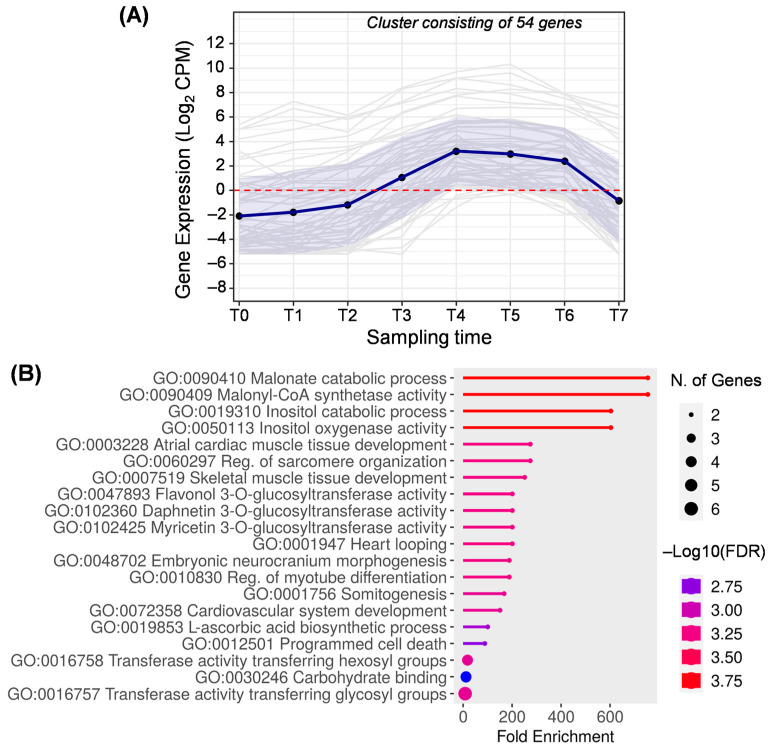
Cluster F. (**A**) Gene expression profile. Blue line represents the mean value of expression for the total genes in the group. Blue shadow represents the standard error of gene expression. Gray lines represent the expression of individual genes. The red horizontal line represents the threshold separating positive from negative expression levels. (**B**) The 20 most representative biological processes according to the FDR and fold enrichment values obtained in ShinyGO 0.80.

Cluster F (Figure 8) includes 54 genes mainly expressed between T3 and T6, that is, from pit hardening to turning purple. This cluster corresponds to cluster 17 generated by DPGP (Figure S1). According to the enrichment analysis, the most characteristic biological processes are those related to malonate metabolism. Specifically, the malonyl–CoA synthetase is the enzyme required for converting free malonate into malonyl–CoA (GO:0090409 and GO:009410). Go terms related to the inositol were enriched in this cluster (GO:0019310 and GO:0050113). Inositol is a precursor of the phytic acid present in the pulp of olive fruit, and its content decreases according to the ripeness [58]. Additionally, phytic acid is necessary for phospholipid formation [1,54].

Genes related to the phenylpropanoids pathway are also present in cluster F due to the activity of the flavonol 3-*O*-glucosyltransferase (GO:0047893). Likewise, terms GO:0102425 and GO:0102360 are obsoleted terms synonymous with flavonol 3-*O*-glucosyltransferase activity (checked at https://www.ebi.ac.uk/QuickGO/, accessed on 24 July 2024).

High activity of the flavonol 3-*O*-glucosyltransferase enzyme in the fruit’s early stages has been observed in previous studies in which this activity concurred with a higher concentration of flavonoids [2,59]. In this cluster, the differential expression of genes involved in flavonoid biosynthesis could be responsible for the fruit veraison [2,49].

Cluster F has the particularity of showing several biological processes related to human health (GO:0003228, GO:0060297, GO:0007519, GO:0001947, GO:0048702, GO:0010830, GO:0072358). Genes included in these biological processes are annotated as RNA-binding proteins (Table S2) that play a key post-transcriptional role in gene expression. In this case, genes specifically coding for rbm38 protein, containing an RRM domain, have been studied in animals, but their role is unknown in plants. Nonetheless, several genes encoding for proteins with the RRM domain for RNA recognition have been described in plants related to the stress response and the flowering time regulation and affecting the fruit ripening process [60].

**Figure 9:**
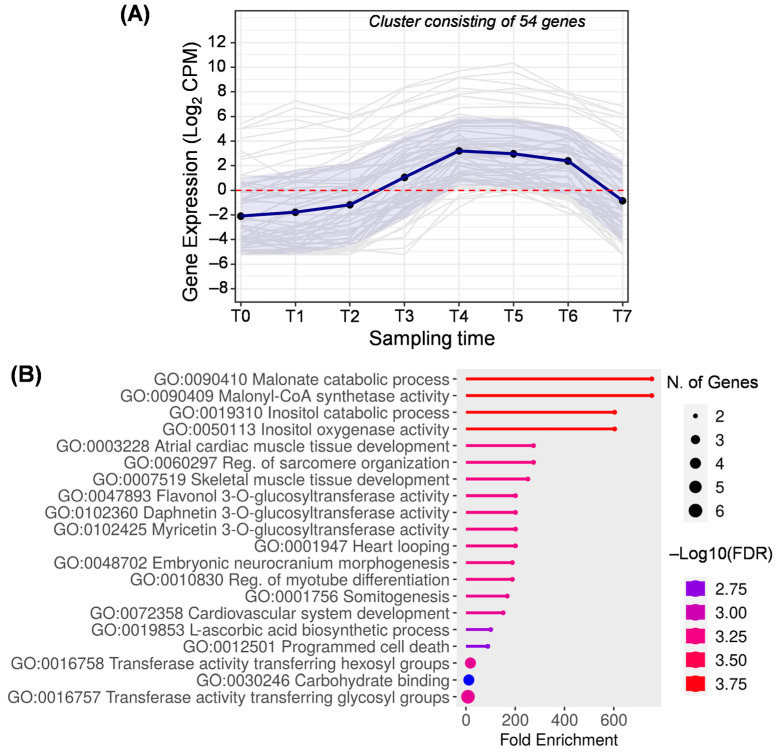
Cluster G. (**A**) Gene expression profile. Blue line represents the mean value of expression for the total genes in the group. Blue shadow represents the standard error of gene expression. Gray lines represent the expression of individual genes. The red horizontal line represents the threshold separating positive from negative expression levels. (**B**) The 20 most representative biological processes according to the FDR and fold enrichment values obtained in ShinyGO 0.80.

Cluster G (Figure 9) encompasses genes with a similar expression profile to clusters E and F (Figure 7 and Figure 8, respectively). The average expression of these genes starts upon pit hardening (similar to cluster F) and follows an increasing trend as fruit ripening progresses (similar to cluster E).

The most enriched biological processes are related to the phyllotactic pattern (GO:0060771, GO:0060772, GO:0060774, and GO:0040019), which could be involved in plant tissue development. However, the annotation of the representative genes of these GO terms revealed *WRI1* genes (*WRINKLED1*) (Table S2). *WRI1* is a key transcription factor that stimulates the expression of many enzymes involved in the conversion of sugar into fatty acids and triacylglycerols during oilseed and oil fruit development [61]. In olive, *WRI* has been described as a key transcription factor in regulating the oil biosynthesis pathway under heat-stress conditions [62].

Other enriched GO terms correspond to fatty acid biosynthetic processes (GO:0006633), and those related to the acetyl–CoA carboxylase enzyme (GO:0009317 and GO:0003989) are especially significant. This biotin-dependent multi-subunit enzyme catalyzes the irreversible carboxylation of acetyl–CoA to produce malonyl–CoA, which represents the first step for the plastidial de novo fatty acid biosynthesis, and it has been demonstrated as a key regulatory point of the pathway.

Other biological processes are also related to fatty acid (GO:0006631) and lipid (GO:0006629) metabolic processes. In addition, cluster G includes specific genes for glycolysis (GO:0004148, GO:0006096, GO:0016668), producing pyruvate [54].

**Figure 10:**
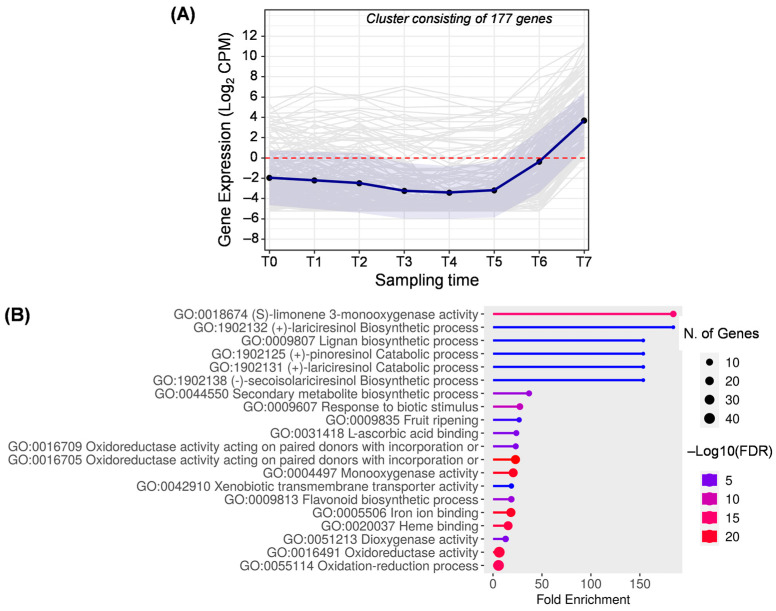
Cluster H. (**A**) Gene expression profile. Blue line represents the mean value of expression for the total genes in the group. Blue shadow represents the standard error of gene expression. Gray lines represent the expression of individual genes. The red horizontal line represents the threshold separating positive from negative expression levels. (**B**) The 20 most representative biological processes according to the FDR and fold enrichment values obtained in ShinyGO 0.80.

Cluster H (Figure 10) encompasses 177 genes, including clusters 7, 8, 23, 28, and 29 from DPGP output. The expression of these genes increases at the late stages of fruit development when the fruit is in its veraison stage until it becomes fully purple (T6–T7).

The most relevant biological processes in this cluster are related to the biosynthesis of secondary metabolites (GO:0044550). Firstly, the formation of terpenes, represented by (S)-limonene 3-monooxygenase activity, is noted. Previous studies described the activity of this enzyme related to the accumulation of limonene at late developmental stages of olive fruit [2,63]. On the other hand, it is noteworthy the representation of genes involved in lignan synthesis, such as those related to the synthesis of (+)-pinoresinol (GO:1902125), (+)-lariciresinol (GO:1902131 and GO:1902132), and (−)-secoisolariciresinol (GO:1902138), all of which are directly related to each other.

The fruit ripening term (GO:0009835) appears for the first time in cluster H, which includes genes coding for polygalacturonase enzymes (Table S2). This finding could suggest the beginning of pectin degradation. In olive, the activity of polygalacturonase has been described on several cultivars, where reduced activity was observed along fruit ripening [64]. As a result of cell wall solubilization, several compounds are released, which act as precursors for ascorbic acid biosynthesis in olive fruits [64]. This fact could also explain the appearance of the biological process GO:0031418.

### 2.2. Identification of Genes Coding for Olive Fatty Acid Desaturase Enzymes

After studying the gene expression during fruit development, it is worth noting the variability of genes involved in the biosynthesis of fatty acids, predominating in almost every cluster. These results support the complexity of fatty acid biosynthesis and their relevance from the very early stages of fruit development. For this reason, this work has focused on identifying genes that code for the fatty acid desaturase enzymes, as well as analyzing their expression levels throughout fruit development. Fatty acid desaturases are key in determining the fatty acid composition of the olive fruit and, therefore, of the olive oil.

As a first step, sequences coding for olive fatty acid desaturase enzymes and isoforms were selected from previous studies, in which their function in fatty acid biosynthesis has been previously validated. Table 1 shows the query and the corresponding sequences in the ‘Picual’ genome *(O. europaea* L. cv. ‘Picual’).

The alignment of the validated gene sequences to the ‘Picual’ genome showed, in some cases, up to two or three homologous genes coding for the same enzyme and isoform. This may be due to DNA duplication events, as noted in previous genome studies [65]. Likewise, the variability in the number of genes involved in oil biosynthesis was observed among cultivars in a wide genome exploration using ‘Ayvalik’, ‘Leccino’, ‘Farga’, and ‘Picual’ [66]. In spite of that, this work provides valuable information about validated genes coding for olive fatty acid desaturase enzymes and isoforms, which is useful for the improvement of the ‘Picual’ genome annotation.

**Figure 11:**
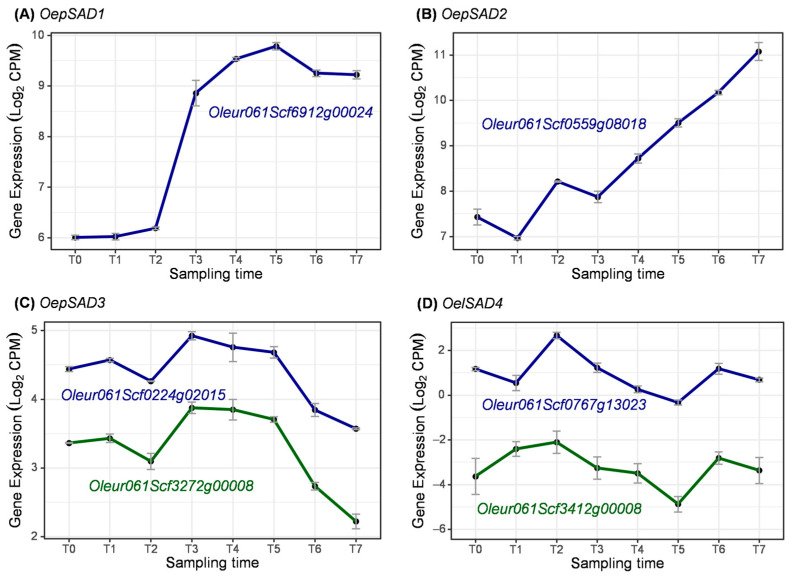
Expression of coding genes for isoforms of SAD enzyme during olive fruit development.

Firstly, stearoyl–ACP desaturase (SAD) is a plastidial soluble enzyme responsible for oleic acid biosynthesis from stearic acid. Figure 11 shows the expression profile of genes coding for SAD isoforms. *OepSAD1* and *OepSAD2* were the most expressed *SAD* genes (Figure 11A,B). Their expressions were increasing as the process of fruit development progressed, with *OepSAD1* transcript levels rising at early stages of fruit development (T1–T3), remaining high at later stages, and those of *OepSAD2* showing a significant increase after pit hardening during the period of oil accumulation (T3–T7). In the same way, *OeSAD1* and *OeSAD2* have previously shown a similar expression pattern by qRT-PCR in the ‘Picual’ and ‘Arbequina’ cultivars [17,67]. These data were further confirmed in ‘Leccino’ and ‘Coratina’ [18], ‘Mari’ and ‘Shengeh’ [68], and in cultivars characterized by extremely low (‘Abou Kanani’) and high (‘Klon-14’) oleic acid content [13].

The two genes coding for *OepSAD3* showed similar expression profiles, decreasing after pit hardening (T3) (Figure 11C). Similarly, low levels of *OeSAD3* gene expression have been reported only in young fruit at early stages of development [15,25,68]. The two genes coding for *OelSAD4* showed low transcript levels and similar expression trends between them (Figure 11D). However, *OeSAD4* expression does not seem to be correlated with oleic acid content in the olive fruit mesocarp [18].

Altogether, unlike the other three olive SAD isoforms, *OeSAD2* exhibits high expression levels in the mesocarp during olive fruit development and ripening, which increased parallel to the unsaturated fatty acid content. Hence, it has been proposed as the gene that contributes mainly to the oleic acid synthesis in the olive mesocarp and, therefore, to its content in the olive oil [15,25].

**Figure 12:**
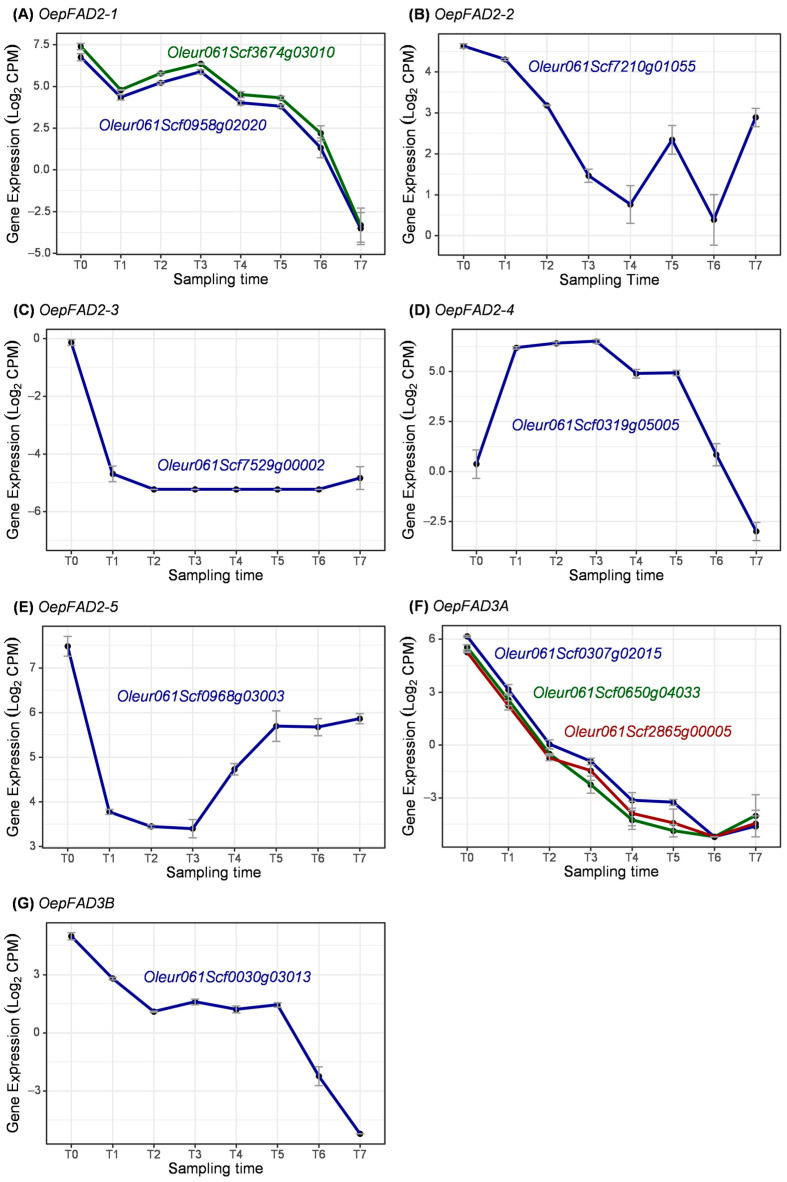
Expression of coding genes for microsomal FAD enzymes during olive fruit development.

Regarding the microsomal enzymes, different isoforms of the oleate desaturase (FAD2) and linoleate desaturase (FAD3) have been studied during fruit development (Figure 12). In this case, genes coding for five different FAD2 isoforms have been identified. A single coding gene has been identified for each isoform, except for *OepFAD2-1*, for which two genes have been identified (Figure 12A), and for *OepFAD3A*, for which there genes were identified (Figure 12F) in the ‘Picual’ genome. For all cases, high levels of expression have been observed for the coding genes of each isoform, except for *OepFAD2-3* (Figure 12C), which has not been expressed at any stage of fruit development.

*FAD2-1* has been described as the main contributor to linoleic acid content in olive seed, whereas *FAD2-2* and *FAD2-5* have been related to the linoleic acid content in the olive mesocarp [20,21,22]. In this work, genes coding for *OepFAD2-1* showed high expression levels from flower until veraison (T6), while the expression of *OepFAD2-2* and *OepFAD2-5* decreased at early developmental stages but increased after pit hardening during the period of oil accumulation (Figure 12B,E). These results are in line with the qRT-PCR expression profile observed for the cultivars ‘Picual’ and ‘Arbequina’ [21,22], ‘Mari’ and ‘Shengeh’ [69], and ‘Leccino’ and ‘Coratina’ [18] and in cultivars characterized by extremely high (‘Abou Kanani’) and low (‘Klon-14’) linoleic acid content [15]. Therefore, these two isoforms (FAD2-2 and FAD2-5) are consistently described as the main responsible for the linoleic acid content in olive fruits and olive oils [15].

In contrast, the role of *OeFAD2-3* and *OeFAD2-4* for the linoleate synthesis in the mesocarp has not been validated [15,22]. Accordingly, in this work, *OepFAD2-3* and *OepFAD2-4* do not increase their transcript levels during the oil accumulation phase (T4–T7) (Figure 12D).

With respect to microsomal linoleate desaturases, *OepFAD3A* and *OepFAD3B* genes, responsible for the production of linolenic acid from linoleic acid, showed high expression at very early stages of fruit development (T1–T3), followed by a sharp decrease after pit hardening, exhibiting very low transcript levels in the mesocarp during the period of oil accumulation (T3–T7) (Figure 12F,G). Similar results have been previously obtained by qRT-PCR in the ‘Picual’ and ‘Arbequina’ cultivars [25]. Consequently, the contribution of *FAD3* genes to the linolenic acid content of the olive mesocarp and, therefore, of the olive oil seems to be very limited. In contrast, *OeFAD3A* has been described as the main gene responsible for the accumulation of linolenic acid in olive seeds due to their contrasting expression profile between mesocarp and seed [25].

**Figure 13:**
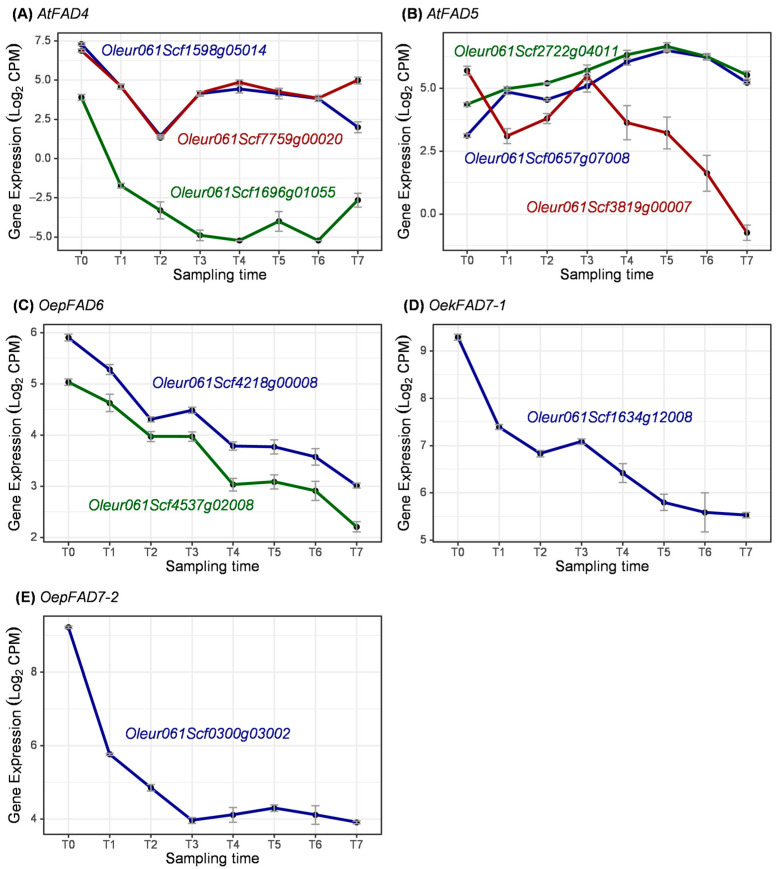
Expression of coding genes for plastidial membrane-bound FAD enzymes during olive fruit development.

Regarding plastidial membrane-bound fatty acid desaturase enzymes, the expression of genes coding for FAD4, FAD5, FAD6, and FAD7 were analyzed throughout fruit development. In all cases, the genes coding for these fatty acid desaturases were highly expressed during fruit development (Figure 13).

FAD4 and FAD5 have not been studied in olive, possibly because they are involved in the fatty acid composition of plastidial lipids and, therefore, not related to the fatty acid profile of the olive oil. Both enzymes are responsible for the desaturation of palmitic acid in the *sn*-2 position of plastidial lipids, with FAD4 acting on phosphatidylglycerol to synthesize trans-palmitoleic acid [27], while FAD5 acts on monogalactosyldiacylglycerol to yield delta-7 palmitoleic acid [28]. Three genes coding for FAD4 were identified in the ‘Picual’ olive genome (Figure 13A). These results are in line with the previous description of FAD genes in the ‘Farga’ cultivar, while the wild olive has only two genes coding for FAD4 [9]. The expression of genes coding for FAD4 was higher in the early stages of fruit development. However, the expression profile of *Oleur061Scf1696g01055* differed from the other two genes coding for this isoform (Figure 13A). Similar results were observed for genes coding for FAD5 (Figure 13B), where the expression pattern of *Oleur061Scf3819g00007* was slightly different from the other genes coding for the same isoform in the ‘Picual’ cultivar. Nevertheless, the expression of *Oleur061Scf2722g04011* and *Oleur061Scf0657g07008* increased from flower (T0) to yellowish fruit (T5) and decreased as the fruit ripened.

FAD6, as well as FAD2, could contribute to the linoleic acid content in olive fruit, although to a lesser extent [15,18,21,69]. The two genes coding for FAD6 showed similar expression patterns decreasing during fruit development (Figure 13C), which is in line with previous studies using qRT-PCR in the cultivars ‘Picual’ and ‘Arbequina’ [21], ‘Leccino’ and ‘Coratina’ [18], and ‘Klon-14’ and ‘Abou Kanani’ [15]. However, the expression pattern of FAD6 genes seems to be cultivar-dependent because of the variability observed during fruit development in these olive cultivars.

Additionally, *OeFAD7-1* and *OeFAD7-2* have a critical role in the linolenic acid content in olive fruit mesocarp [25]. The role of *OeFAD7-1* has been characterized in the ‘Koroneiki’ [26], ‘Picual’, and ‘Arbequina’ cultivars [25], whereas *OeFAD7-2* has been validated in the ‘Picual’ and ‘Arbequina’ cultivars [25]. In this work, single genes were identified coding for each FAD7 isoform, and their transcripts decreased during fruit development mainly in early stages (Figure 13D,E). On the contrary, the expression pattern in the ‘Picual’ and ‘Arbequina’ [25] and ‘Klon-14’ and ‘Abou Kanani’ cultivars [15] showed a constant transcript level for both genes during mesocarp development and ripening.

## 3. Materials and Methods

### 3.1. RNAseq Data and Transcriptomic Analysis

RNAseq data from the bioproject number PRJNA870905, available in NCBI database, was used for this study. Specifically, the data correspond to flower and fruit samples of the olive cultivar ‘Picual’ collected from three different trees on south-facing branches. Sampling was performed at seven different times, from full blooming (T0: flower) to full maturity, including fruit at 15 days after full blooming (AFB) (T1: embryo’s growth) and monthly until 6 months AFB (T2: young drupe, T3: pit hardened, T4: green, T5: yellowish, T6: veraison, T7: completely purple). The samples were stored at −80 °C until processing.

RNA extraction from full samples (T0 to T3) or from mesocarp (T4 to T7) was performed using the Spectrum™ Plant kit (Merck KGaA, Darmstadt, Germany). After purification, strand-specific sequencing was performed with the NGS Illumina pair-end technology (150 bp × 2) by the company Sistemas Genómicos S.L. (Paterna, Valencia, Spain).

For transcriptomic analysis, STAR v2.7 [70] was used to align reads to the reference genome of ‘Picual’ [65], and aligned fragments were counted with featureCounts v2.0.6 [71]. The differentially expressed genes were identified by applying EdgeR v4.2 [72], comparing any stage of the fruit development process (T1–T7) to the flowering stage (T0). To consider a gene differentially expressed, values of *p*-adj < 0.01 and fold change > 4 or <−4 were set. Overexpressed genes were annotated using Sma3s v2 [73]. Afterward, the expression profile of those genes overexpressing at any point AFB (i.e., T1–T7) was analyzed using DPGP v0.1 [29] by clustering those showing a similar expression pattern. The normalized CPM values extracted from EdgeR v4.2 were used as input for DPGP v0.1. The clusters generated by DPGP v0.1 showing similar expression profiles were regrouped and plotted using the ggplot2 package v3.4.4 in Rstudio v4.2.1. These final clusters were subjected to gene enrichment analysis for biological processes using ShinyGO v0.8 web tool (http://bioinformatics.sdstate.edu/go/ (accessed on 21 June 2024)) [74]. For this analysis, the total of differentially expressed genes, both up and down, as background, were considered as background.

### 3.2. Fatty Acid Desaturase Gene Identification

In this study, special emphasis has been given to those genes involved in fatty acid desaturation. For this purpose, sequences of genes coding for fatty acid desaturases were searched on the OliveTreeDB database (https://genomaolivar.dipujaen.es/db/index.php (accessed on 2 April 2024)). Specifically, homologous sequences to *OepSAD1*, *OepSAD2* and *OepSAD3* [17], *OelSAD4* [18], *OepFAD2-1*, *OepFAD2-2*, *OepFAD2-3*, *OepFAD2-4* and *OepFAD2-5* [20,22], *OepFAD3A* and *OepFAD3B*, [25] *AtFAD4* [27], *AtFAD5* [28], *OepFAD6* [23], *OekFAD7-1* [26], and *OepFAD7-2* [25] were searched based on their description and validation in previous works.

## 4. Conclusions

This work represents an effort to characterize the transcriptional profile throughout fruit development, including in several stages from flowering (full blooming) until fruit ripening (6 months AFB). The objective is to characterize the importance of those genes overexpressed during olive fruit development.

In general, a decay of gene expression has been observed throughout fruit development. Therefore, the inclusion of samples close in time to the early stages of fruit development has provided novel information about the processes occurring at that time, which have usually been ignored in most of the work published so far.

This work has highlighted eight clusters of genes showing different expression profiles. At the beginning of fruit development (T1, cluster A), hormones played an important role, together with the synthesis of cellulose as the primary component of the cell wall and the accumulation of sugar compounds before pit hardening (T2, cluster B). Genes involved in the synthesis of phenolic compounds were detected from the beginning of fruit development (T2 and T3, cluster C), maintaining their relevance throughout the whole process of fruit development. Nonetheless, the higher expression of genes involved in the synthesis of fatty acids was observed at intermediate stages (clusters E and G), even increasing as the fruit ripens. In the end, when the fruit turns completely purple (T6–T7, cluster H), cell wall degradation processes appear.

Gene expression analysis has corroborated the complexity of the fatty acid synthesis process and the variability of genes involved in this process throughout olive fruit development. Therefore, a detailed analysis of validated genes coding for the fatty acid desaturase enzymes responsible for the fatty acid composition has been carried out. The identification of genes coding for *SAD*, *FAD2*, *FAD3*, *FAD4*, *FAD5*, *FAD6*, and *FAD7* in the ‘Picual’ genome has improved the genome annotation. Moreover, the expression analysis of these genes has corroborated their role in the synthesis of fatty acids in olive fruits, except for the genes coding for *OeSAD4* and *OeFAD2-3*, which showed negligible expression throughout the fruit development.

## Figures and Tables

**Table 1:** Genes coding for olive fatty acid desaturase enzymes and isoforms.

Characterized Gene (Query)			Blast Results		
Gene	Accession Number	Reference	Homologue in Picual Genome	Score (Bits)	E-Value
*OepSAD1*	KX196198	[17]	*Oleur061Scf6912g00024.1*	767	0
*OepSAD2*	KX196199	[17]	*Oleur061Scf0559g08018.1*	590	0
*OepSAD3*	KX196200	[17]	*Oleur061Scf0224g02015.1*	831	0
			*Oleur061Scf3272g00008.1*	802	0
*OelSAD4*	MN821530	[18]	*Oleur061Scf0767g13023.1*	836	0
			*Oleur061Scf3412g00008.1*	695	0
*OepFAD2-1*	AY733076	[20]	*Oleur061Scf3674g03010.1*	786	0
			*Oleur061Scf0958g02020.1*	780	0
*OepFAD2-2*	AY733077	[20]	*Oleur061Scf7210g01055.1*	793	0
*OepFAD2-3*	MN103339	[22]	*Oleur061Scf7529g00002.1*	261	2 × 10^−87^
*OepFAD2-4*	MN103340	[22]	*Oleur061Scf0319g05005.1*	687	0
*OepFAD2-5*	MN103341	[22]	*Oleur061Scf0968g03003.1*	794	0
*OepFAD3A*	KP893692	[25]	*Oleur061Scf0307g02015.1*	253	2 × 10^−83^
			*Oleur061Scf0650g04033.1*	252	1 × 10^−82^
			*Oleur061Scf2865g00005.1*	210	8 × 10^−68^
*OepFAD3B*	KP893693	[25]	*Oleur061Scf0030g03013.1*	297	1 × 10^−100^
*AtFAD4*	AT4G27030	[27]	*Oleur061Scf7759g00020.1*	385	1 × 10^−134^
			*Oleur061Scf1598g05014.1*	383	8 × 10^−134^
			*Oleur061Scf1696g01055.1*	283	1 × 10^−95^
*AtFAD5*	AT2G31360	[28]	*Oleur061Scf0657g07008.1*	356	2 × 10^−122^
			*Oleur061Scf2722g04011.1*	307	3 × 10^−123^
			*Oleur061Scf3819g00007.1*	260	3 × 10^−85^
*OepFAD6*	AY733075	[23]	*Oleur061Scf4218g00008.1*	898	0
			*Oleur061Scf4537g02008.1*	803	0
*OekFAD7-1*	DQ788674	[26]	*Oleur061Scf1634g12008.1*	380	2 × 10^−131^
*OepFAD7-2*	KP893695	[25]	*Oleur061Scf0300g03002.1*	769	0

## Data Availability

Data is contained within the article and Supplementary Materials.

## Supplementary Materials

The supporting information can be downloaded at: https://www.mdpi.com/article/10.3390/ijms252011150/s1.

## References

[1] Conde C., Delrot S., Gerós H. (2008). Physiological, Biochemical and Molecular Changes Occurring during Olive Development and Ripening. J. Plant Physiol..

[2] Galla G., Barcaccia G., Ramina A., Collani S., Alagna F., Baldoni L., Cultrera N.G., Martinelli F., Sebastiani L., Tonutti P. (2009). Computational Annotation of Genes Differentially Expressed along Olive Fruit Development. BMC Plant Biol..

[3] Camarero M.C., Briegas B., Corbacho J., Labrador J., Gallardo M., Gomez-Jimenez M.C. (2023). Characterization of Transcriptome Dynamics during Early Fruit Development in Olive (*Olea europaea* L.). Int. J. Mol. Sci..

[4] Hammami S.B.M., Costagli G., Rapoport H.F. (2013). Cell and Tissue Dynamics of Olive Endocarp Sclerification Vary According to Water Availability. Physiol. Plant..

[5] Ghanbari R., Anwar F., Alkharfy K.M., Gilani A.-H., Saari N. (2012). Valuable Nutrients and Functional Bioactives in Different Parts of Olive (*Olea europaea* L.)—A Review. Int. J. Mol. Sci..

[6] Skodra C., Titeli V.S., Michailidis M., Bazakos C., Ganopoulos I., Molassiotis A., Tanou G. (2021). Olive Fruit Development and Ripening: Break on through to the “-Omics” Side. Int. J. Mol. Sci..

[7] Unver T., Wu Z., Sterck L., Turktas M., Lohaus R., Li Z., Yang M., He L., Deng T., Escalante F.J. (2017). Genome of Wild Olive and the Evolution of Oil Biosynthesis. Proc. Natl. Acad. Sci. USA.

[8] Liu X., Guo L., Zhang J., Xue L., Luo Y., Rao G. (2021). Integrated Analysis of Fatty Acid Metabolism and Transcriptome Involved in Olive Fruit Development to Improve Oil Composition. Forests.

[9] Niu E., Gao S., Hu W., Zhang C., Liu D., Shen G., Zhu S. (2022). Genome-Wide Identification and Functional Differentiation of Fatty Acid Desaturase Genes in *Olea europaea* L.. Plants.

[10] Gil-Amado J.A., Gomez-Jimenez M.C. (2013). Transcriptome Analysis of Mature Fruit Abscission Control in Olive. Plant Cell Physiol.

[11] Parra R., Paredes M.A., Sanchez-Calle I.M., Gomez-Jimenez M.C. (2013). Comparative Transcriptional Profiling Analysis of Olive Ripe-Fruit Pericarp and Abscission Zone Tissues Shows Expression Differences and Distinct Patterns of Transcriptional Regulation. BMC Genom..

[12] Briegas B., Corbacho J., Parra-Lobato M.C., Paredes M.A., Labrador J., Gallardo M., Gomez-Jimenez M.C. (2020). Transcriptome and Hormone Analyses Revealed Insights into Hormonal and Vesicle Trafficking Regulation among *Olea europaea* Fruit Tissues in Late Development. Int. J. Mol. Sci..

[13] Camarero M.C., Briegas B., Corbacho J., Labrador J., Gomez-Jimenez M.C. (2023). Hormonal Content and Gene Expression during Olive Fruit Growth and Ripening. Plants.

[14] Bodoira R., Torres M., Pierantozzi P., Taticchi A., Servili M., Maestri D. (2015). Oil Biogenesis and Antioxidant Compounds from “Arauco” Olive (*Olea europaea* L.) Cultivar during Fruit Development and Ripening. Eur. J. Lipid Sci. Technol..

[15] Hernández M.L., Sicardo M.D., Belaj A., Martínez-Rivas J.M. (2021). The Oleic/Linoleic Acid Ratio in Olive (*Olea europaea* L.) Fruit Mesocarp Is Mainly Controlled by OeFAD2-2 and OeFAD2-5 Genes Together With the Different Specificity of Extraplastidial Acyltransferase Enzymes. Front. Plant Sci..

[16] Haralampidis K., Milioni D., Sánchez J., Baltrusch M., Heinz E., Hatzopoulos P. (1998). Temporal and Transient Expression of Stearoyl-ACP Carrier Protein Desaturase Gene during Olive Fruit Development. J. Exp. Bot..

[17] Parvini F., Sicardo M.D., Hosseini-Mazinani M., Martínez-Rivas J.M., Hernández M.L. (2016). Transcriptional Analysis of Stearoyl-Acyl Carrier Protein Desaturase Genes from Olive (*Olea europaea*) in Relation to the Oleic Acid Content of the Virgin Olive Oil. J. Agric. Food Chem..

[18] Contreras C., Mariotti R., Mousavi S., Baldoni L., Guerrero C., Roka L., Cultrera N., Pierantozzi P., Maestri D., Gentili L. (2020). Characterization and Validation of Olive *FAD* and *SAD* Gene Families: Expression Analysis in Different Tissues and during Fruit Development. Mol. Biol. Rep..

[19] Banilas G., Moressis A., Nikoloudakis N., Hatzopoulos P. (2005). Spatial and Temporal Expressions of Two Distinct Oleate Desaturases from Olive (*Olea europaea* L.). Plant Sci..

[20] Hernández M.L., Mancha M., Martínez-Rivas J.M. (2005). Molecular Cloning and Characterization of Genes Encoding Two Microsomal Oleate Desaturases (*FAD2*) from Olive. Phytochemistry.

[21] Hernández M.L., Padilla M.N., Mancha M., Martínez-Rivas J.M. (2009). Expression Analysis Identifies *FAD2-2* as the Olive Oleate Desaturase Gene Mainly Responsible for the Linoleic Acid Content in Virgin Olive Oil. J. Agric. Food Chem..

[22] Hernández M.L., Sicardo M.D., Arjona P.M., Martínez-Rivas J.M. (2020). Specialized Functions of Olive *FAD2* Gene Family Members Related to Fruit Development and the Abiotic Stress Response. Plant Cell Physiol..

[23] Hernández M.L., Padilla M.N., Sicardo M.D., Mancha M., Martínez-Rivas J.M. (2011). Effect of Different Environmental Stresses on the Expression of Oleate Desaturase Genes and Fatty Acid Composition in Olive Fruit. Phytochemistry.

[24] Banilas G., Nikiforiadis A., Makariti I., Moressis A., Hatzopoulos P. (2007). Discrete Roles of a Microsomal Linoleate Desaturase Gene in Olive Identified by Spatiotemporal Transcriptional Analysis. Tree Physiol..

[25] Hernández M.L., Sicardo M.D., Martínez-Rivas J.M. (2016). Differential Contribution of Endoplasmic Reticulum and Chloroplast ω-3 Fatty Acid Desaturase Genes to the Linolenic Acid Content of Olive (*Olea europaea*) Fruit. Plant Cell Physiol..

[26] Poghosyan Z.P., Haralampidis K., Martsinkovskaya A.I., Murphy D.J., Hatzopoulos P. (1999). Developmental Regulation and Spatial Expression of a Plastidial Fatty Acid Desaturase from *Olea europaea*. Plant Physiol. Biochem..

[27] Gao J., Ajjawi I., Manoli A., Sawin A., Xu C., Froehlich J.E., Last R.L., Benning C. (2009). *FATTY ACID DESATURASE4* of Arabidopsis Encodes a Protein Distinct from Characterized Fatty Acid Desaturases. Plant J..

[28] Heilmann I., Mekhedov S., King B., Browse J., Shanklin J. (2004). Identification of the Arabidopsis Palmitoyl-Monogalactosyldiacylglycerol Δ7-Desaturase Gene *FAD5*, and Effects of Plastidial Retargeting of Arabidopsis Desaturases on the *Fad5* Mutant Phenotype. Plant Physiol..

[29] McDowell I.C., Manandhar D., Vockley C.M., Schmid A.K., Reddy T.E., Engelhardt B.E. (2018). Clustering Gene Expression Time Series Data Using an Infinite Gaussian Process Mixture Model. PLoS Comput. Biol..

[30] Brzycki Newton C., Young E.M., Roberts S.C. (2023). Targeted Control of Supporting Pathways in Paclitaxel Biosynthesis with CRISPR-Guided Methylation. Front. Bioeng. Biotechnol..

[31] García-Vico L., Belaj A., Sánchez-Ortiz A., Martínez-Rivas J., Pérez A., Sanz C. (2017). Volatile Compound Profiling by HS-SPME/GC-MS-FID of a Core Olive Cultivar Collection as a Tool for Aroma Improvement of Virgin Olive Oil. Molecules.

[32] Ouni Y., Flamini G., Zarrouk M. (2016). The Chemical Properties and Volatile Compounds of Virgin Olive Oil from Oueslati Variety: Influence of Maturity Stages in Olives. J. Am. Oil Chem. Soc..

[33] Vezzaro A., Krause S.T., Nonis A., Ramina A., Degenhardt J., Ruperti B. (2012). Isolation and Characterization of Terpene Synthases Potentially Involved in Flavor Development of Ripening Olive (*Olea europaea*) Fruits. J. Plant Physiol..

[34] Fenn M.A., Giovannoni J.J. (2021). Phytohormones in Fruit Development and Maturation. Plant J..

[35] Matsuo S., Kikuchi K., Nagasuga K., Ueno H., Imanishi S. (2018). Transcriptional Regulation of Auxin Metabolic-Enzyme Genes during Tomato Fruit Development. Sci. Hortic..

[36] Zheng L., Zhang L., Duan K., Zhu Z.-P., Ye Z.-W., Gao Q.-H. (2016). *YUCCA* Type Auxin Biosynthesis Genes Encoding Flavin Monooxygenases in Melon: Genome-Wide Identification and Developmental Expression Analysis. South Afr. J. Bot..

[37] Eroglu S., Giehl R.F.H., Meier B., Takahashi M., Terada Y., Ignatyev K., Andresen E., Küpper H., Peiter E., von Wirén N. (2017). Metal Tolerance Protein 8 Mediates Manganese Homeostasis and Iron Reallocation during Seed Development and Germination. Plant Physiol..

[38] Cirilli M., Caruso G., Gennai C., Urbani S., Frioni E., Ruzzi M., Servili M., Gucci R., Poerio E., Muleo R. (2017). The Role of Polyphenoloxidase, Peroxidase, and β-Glucosidase in Phenolics Accumulation in *Olea europaea* L. Fruits under Different Water Regimes. Front. Plant Sci..

[39] Adamski N.M., Anastasiou E., Eriksson S., O’Neill C.M., Lenhard M. (2009). Local Maternal Control of Seed Size by KLUH/CYP78A5-Dependent Growth Signaling. Proc. Natl. Acad. Sci. USA.

[40] Perez-Arcoiza A., Luisa Hernández M., Dolores Sicardo M., Hernandez-Santana V., Diaz-Espejo A., Martinez-Rivas J.M. (2022). Carbon Supply and Water Status Regulate Fatty Acid and Triacylglycerol Biosynthesis at Transcriptional Level in the Olive Mesocarp. Plant Cell Environ..

[41] Ben-Ari G., Biton I., Many Y., Namdar D., Samach A. (2021). Elevated Temperatures Negatively Affect Olive Productive Cycle and Oil Quality. Agronomy.

[42] Benlloch-González M., Sánchez-Lucas R., Bejaoui M.A., Benlloch M., Fernández-Escobar R. (2019). Global Warming Effects on Yield and Fruit Maturation of Olive Trees Growing under Field Conditions. Sci. Hortic..

[43] Vierhuis E., Schols H.A., Beldman G., Voragen A.G.J. (2000). Isolation and Characterisation of Cell Wall Material from Olive Fruit (*Olea europaea* Cv Koroneiki) at Different Ripening Stages. Carbohydr. Polym..

[44] Jiménez A., Rodríguez R., Fernández-Caro I., Guillén R., Fernández-Bolaños J., Heredia A. (2001). Olive Fruit Cell Wall:  Degradation of Cellulosic and Hemicellulosic Polysaccharides during Ripening. J. Agric. Food Chem..

[45] Parra R., Gomez-Jimenez M.C. (2020). Spatio–Temporal Immunolocalization of Extensin Protein and Hemicellulose Polysaccharides during Olive Fruit Abscission. Planta.

[46] Seifi E., Guerin J., Kaiser B., Sedgley M. (2015). Flowering and fruit set in olive: A review. Iran. J. Plant Physiol..

[47] Alagna F., D’Agostino N., Torchia L., Servili M., Rao R., Pietrella M., Giuliano G., Chiusano M.L., Baldoni L., Perrotta G. (2009). Comparative 454 Pyrosequencing of Transcripts from Two Olive Genotypes during Fruit Development. BMC Genom..

[48] Khan M.K.U., Muhammad N., Jia Z., Peng J., Liu M. (2022). Mechanism of Stone (Hardened Endocarp) Formation in Fruits: An Attempt toward Pitless Fruits, and Its Advantages and Disadvantages. Genes.

[49] Bianco L., Alagna F., Baldoni L., Finnie C., Svensson B., Perrotta G. (2013). Proteome Regulation during *Olea europaea* Fruit Development. PLoS ONE.

[50] Zhang D., Ji K., Wang J., Liu X., Zhou Z., Huang R., Ai G., Li Y., Wang X., Wang T. (2024). Nuclear Factor Y-A3b Binds to the SINGLE FLOWER TRUSS Promoter and Regulates Flowering Time in Tomato. Hortic. Res..

[51] Laloum T., De Mita S., Gamas P., Baudin M., Niebel A. (2013). CCAAT-Box Binding Transcription Factors in Plants: Y so Many?. Trends Plant Sci..

[52] Padilla M.N., Hernández M.L., Sanz C., Martínez-Rivas J.M. (2009). Functional Characterization of Two 13-Lipoxygenase Genes from Olive Fruit in Relation to the Biosynthesis of Volatile Compounds of Virgin Olive Oil. J. Agric. Food Chem..

[53] Aguirrebengoa M., Moreno B., Alcalá-Herrera R., Núñez R., Guirado N., García J.M., Pozo M.J., Benítez E. (2024). Modulation of Volatile Emissions in Olive Trees: Sustained Effect of Trichoderma Afroharzianum T22 on Induced Plant Defenses after Simulated Herbivory. Biol. Fertil. Soils.

[54] Salas J.J., Harwood J.L., Martínez-Force E., Aparicio R., Harwood J. (2013). Lipid Metabolism in Olive: Biosynthesis of Triacylglycerols and Aroma Components. Handbook of Olive Oil: Analysis and Properties.

[55] Broz A.K., Tovar-Méndez A., Mooney B.P., Johnston M.L., Miernyk J.A., Randall D.D. (2014). A Novel Regulatory Mechanism Based upon a Dynamic Core Structure for the Mitochondrial Pyruvate Dehydrogenase Complex?. Mitochondrion.

[56] Garrido A., Conde A., Serôdio J., De Vos R.C.H., Cunha A. (2023). Fruit Photosynthesis: More to Know about Where, How and Why. Plants.

[57] Poghosyan Z.P., Giannoulia K., Katinakis P., Murphy D.J., Hatzopoulos P. (2005). Temporal and Transient Expression of Olive Enoyl-ACP Reductase Gene during Flower and Fruit Development. Plant Physiol. Biochem..

[58] Marsilio V., Campestre C., Lanza B., De Angelis M. (2001). Sugar and Polyol Compositions of Some European Olive Fruit Varieties (*Olea europaea* L.) Suitable for Table Olive Purposes. Food Chem..

[59] Martinelli F., Tonutti P. (2012). Flavonoid Metabolism and Gene Expression in Developing Olive (*Olea europaea* L.) Fruit. Plant Biosyst. Int. J. Deal. All Asp. Plant Biol..

[60] Ma L., Cheng K., Li J., Deng Z., Zhang C., Zhu H. (2021). Roles of Plant Glycine-Rich RNA-Binding Proteins in Development and Stress Responses. Int. J. Mol. Sci..

[61] Kuczynski C., McCorkle S., Keereetaweep J., Shanklin J., Schwender J. (2022). An Expanded Role for the Transcription Factor WRINKLED1 in the Biosynthesis of Triacylglycerols during Seed Development. Front. Plant Sci..

[62] Nissim Y., Shlosberg M., Biton I., Many Y., Doron-Faigenboim A., Hovav R., Kerem Z., Avidan B., Ben-Ari G. (2020). A High Temperature Environment Regulates the Olive Oil Biosynthesis Network. Plants.

[63] Alagna F., Mariotti R., Panara F., Caporali S., Urbani S., Veneziani G., Esposto S., Taticchi A., Rosati A., Rao R. (2012). Olive Phenolic Compounds: Metabolic and Transcriptional Profiling during Fruit Development. BMC Plant Biol..

[64] Diarte C., Iglesias A., Romero A., Casero T., Ninot A., Gatius F., Graell J., Lara I. (2021). Ripening-Related Cell Wall Modifications in Olive (*Olea europaea* L.) Fruit: A Survey of Nine Genotypes. Food Chem..

[65] Jiménez-Ruiz J., Ramírez-Tejero J.A., Fernández-Pozo N., Leyva-Pérez M.d.l.O., Yan H., de la Rosa R., Belaj A., Montes E., Rodríguez-Ariza M.O., Navarro F. (2020). Transposon Activation Is a Major Driver in the Genome Evolution of Cultivated Olive Trees (*Olea europaea* L.). Plant Genome.

[66] Vatansever R., Hernandez P., Escalante F.J., Dorado G., Unver T. (2022). Genome-Wide Exploration of Oil Biosynthesis Genes in Cultivated Olive Tree Varieties (*Olea europaea*): Insights into Regulation of Oil Biosynthesis. Funct. Integr. Genom..

[67] Hernández M.L., Sicardo M.D., Alfonso M., Martínez-Rivas J.M. (2019). Transcriptional Regulation of Stearoyl-Acyl Carrier Protein Desaturase Genes in Response to Abiotic Stresses Leads to Changes in the Unsaturated Fatty Acids Composition of Olive Mesocarp. Front. Plant Sci..

[68] Razeghi-Jahromi F., Parvini F., Zarei A., Hosseini-Mazinani M. (2022). Sequence Characterization and Temporal Expression Analysis of Different *SADs* and *FAD2-2* Genes in Two Iranian Olive Cultivars. Sci. Hortic..

[69] Parvini F., Zeinanloo A.A., Ebrahimie E., Tahmasebi-Enferadi S., Hosseini-Mazinani M. (2015). Differential Expression of Fatty Acid Desaturases in Mari and Shengeh Olive Cultivars during Fruit Development and Ripening. Eur. J. Lipid Sci. Technol..

[70] Dobin A., Davis C.A., Schlesinger F., Drenkow J., Zaleski C., Jha S., Batut P., Chaisson M., Gingeras T.R. (2013). STAR: Ultrafast Universal RNA-Seq Aligner. Bioinformatics.

[71] Liao Y., Smyth G.K., Shi W. (2014). featureCounts: An Efficient General Purpose Program for Assigning Sequence Reads to Genomic Features. Bioinformatics.

[72] Robinson M.D., McCarthy D.J., Smyth G.K. (2010). edgeR: A Bioconductor Package for Differential Expression Analysis of Digital Gene Expression Data. Bioinformatics.

[73] Casimiro-Soriguer C.S., Muñoz-Mérida A., Pérez-Pulido A.J. (2017). Sma3s: A Universal Tool for Easy Functional Annotation of Proteomes and Transcriptomes. Proteomics.

[74] Ge S.X., Jung D., Yao R. (2020). ShinyGO: A Graphical Gene-Set Enrichment Tool for Animals and Plants. Bioinformatics.

