# Supplementary material for: Transcriptomic Analysis During Olive Fruit Development and Expression Profiling of Fatty Acid Desaturase Genes
Source: Int J Mol Sci. 2024 Oct 17;25(20):11150. doi: 10.3390/ijms252011150 (PMC11508905; doi:10.3390/ijms252011150)
Supplement: Supplementary file 1 [file ijms-25-11150-s001.zip › Supplementary data/ijms-3263884-supplementary.pdf]

Figure S1: Clusters of genes showing similar expression pattern obtained by DPGP software;

Table S1: Quality of sequencing data and alignment ratios;

Table S2: List of genes included into each cluster and its annotation results according Sma3s;

Table S3: Enrichment analysis results obtained from ShinyGO 0.8.
